# Mortality Statistics of Buffalo

**Published:** 1874-04

**Authors:** 


### Mortality Statistics of Buffalo.

We have received the Official Report of death in the City of Buffalo for the year 1873. The statements therein contained present some interesting and suggestive facts.

The total number of deaths from all causes was 2336. In the report the population is estimated at 170,000, which would give a death ratio of 13.73 per thousand. Perhaps the estimated population may be considered by some as a little above the mark; probably none, however, will be willing to admit a population of less than 150,000 for the city, this would give us a death rate of 15.57 per thousand. Our population is estimated by competent judges as far above this figure however; but that we may not be accused of claiming too much for the health of the city, we place it at this limit. It will be seen that by this estimate even we compare favorably with other cities. We have have been unable to obtain the reports for 1873, bnt make the comparisons from those of 1872. The highest death rate in 1872, was at Memphis, where it reached 46.6 per thousand. Then comes in the following order Savannah, 39.2, Vicksbure, 36.5, Troy, 34, Hoboken, 32.9, New York, 32 07 New Orleans, 30.6, Boston, 30.5, Philadelphia, 26.1, Baltimore, 25.1, Cincinnati, 20.5, St. Louis, 20.1, San Francisco, 17.3.

The disease which exhibits the greatest mortality in the city for 1873, is Consumption, there being 243 deaths reported from it. Next in order comes Cholera Infantum, from which 232 deaths are reported. Convulsions are reported next in order, there being 229 fatal cases, and from Inflammation of the Lungs 145 cases. Typhoid Fever presents a mortality of 96, none of the other diseases reaching above 90. Of the whole number of deaths no cause is assigned to 121 cases. The condition in life of those who died shows a high rate of mortality among those who enjoy a state of single blessedness; 618 were married, 1353 single, 118 widows, 77 widowers and 170 whose condition is not given. The causes of death were certified to by regular physicians including public institutions and the city at large in 1246 cases. By irregular practitioners in 363 cases, by Coroners and Undertakers 727 instances. It being fair to presume that the ratio of deaths to the number of cases treated is equal among irregular practitioners, (which includes all who practice medicine not members of Erie County Medical Society,) to that among regular physicians, this report shows that the "quacks" do not get as large a share of public patronage as they would have us suppose.

